# A new species of *Orobdella* (Hirudinida, Arhynchobdellida, Gastrostomobdellidae) and redescription of *Orobdella kawakatsuorum* from Hokkaido, Japan with the phylogenetic position of the new species

**DOI:** 10.3897/zookeys.169.2425

**Published:** 2012-02-10

**Authors:** Takafumi Nakano

**Affiliations:** 1 Department of Zoology, Graduate School of Science, Kyoto University, Kyoto 606-8502, Japan

**Keywords:** Hirudinida, Hirudinea, Gastrostomobdellidae, *Orobdella kawakatsuorum*, new species, molecular phylogeny, Japan

## Abstract

A new quadrannulate *Orobdella* Oka, 1895 species, *Orobdella koikei* **sp. n.**, is described on the basis of six specimens collected from Hokkaido, Japan. In addition, an emended description of quadrannulate *Orobdella kawakatsuorum* Richardson, 1975 is also provided. *Orobdella koikei* differs from other quadrannulate species of *Orobdella* in possessing the following combination of characters: color dorsally brown, IV uniannulate, male gonopore at XI b6, gastropore and female gonopore at XIII a1, 1/2 + 4 + 1/2 between gonopores, XXV triannulate, tubular but bulbous at junctions with gastropore and crop gastroporal duct, epididymides in XVII to XIX, and atrial cornua ovate. The phylogenetic position of the newly described species is estimated using mitochondrial COI, tRNA^Cys^, tRNA^Met^, 12S rDNA, tRNA^Val^ and 16S rDNA markers. *Orobdella koikei* is a sister taxon of *Orobdella kakawatsuorum* according to the molecular phylogenetic analyses.

## Introduction

The genus *Orobdella* Oka, 1895 consists of terrestrial gastroporous leeches in East Asia (Sawyer 1986). The species diversity of *Orobdella* has been revised recently, and now this genus includes eight species (Nakano 2010, 2011a, b). Among these species, only one quadrannulate species, *Orobdella kawakatsuorum* Richardson, 1975, has been known from Hokkaido, Japan (Richardson 1975). This species was described based on the two specimens collected from Sapporo, and its holotype has been deposited at the National Museum of Nature and Science, Tokyo (NSMT). *Orobdella kawakatsuorum* is characterized especially by its possession of six annuli between gonopores and a simple tubular gastroporal duct.

Quadrannulate *Orobdella* specimens were recently obtained from various places in Hokkaido. Most of these specimens were identified as *Orobdella kawakatsuorum*. However, several specimens differ from not only *Orobdella kawakatsuorum*, but also the other quadrannulate species, *Orobdella esulcata* Nakano, 2010, *Orobdella tsushimensis* Nakano, 2011, and *Orobdella whitmani* Oka, 1895, in several characterisics. Therefore, they are described as a new species herein. In addition, an emended description of *Orobdella kawakatsuorum* is presented on the basis of its holotype and newly collected materials. The phylogenetic position of the new species is also estimated using mitochondrial COI, tRNA^Cys^, tRNA^Met^, 12S rDNA, tRNA^Val^ and 16S rDNA sequence data.

## Materials and methods

**Figure 1.**
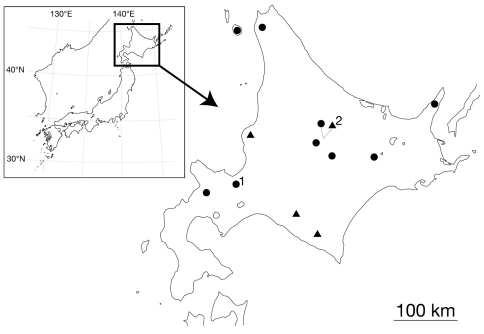
Map showing the collection localities of *Orobdella koikei* sp. n. and *Orobdella kawakatsuorum* Richardson, 1975. Black triangles indicate the localities of *Orobdella koikei*; black circles indicate those of *Orobdella kawakatsuorum* **1** type locality of *Orobdella kawakatsuorum*; and **2** type locality of *Orobdella koikei*.

For the taxonomic study, leeches were collected from Hokkaido, Japan (Fig. 1), under rocks along mountain or forest trails. Altitude and coordinates for localities were obtained using a Garmin eTrex GPS unit.

 The preparation of the collected materials for the morphological and molecular analyses follows Nakano (2011b). Two measurements were taken: body length (BL) from the anterior margin of the oral sucker to the posterior margin of the caudal sucker, and maximum body width (BW). Examination, dissection, and drawing of the specimens were accomplished under stereoscopic microscopes with drawing tubes (Leica S6E, M125 and WILD HEERBRUGG TYP 308700).

The numbering convention is based on Moore (1927): body somites are denoted by Roman numerals and annuli in each somite are given alphanumeric designations.

For the molecular phylogenetic analyses, the sequence data of nine *Orobdella* species were newly obtained (Table 1). As outgroup, three Erpobdelliformes leeches, *Gastrostomobdella monticola* Moore, 1929 (Gastrostomobdellidae), *Erpobdella japonica* Pawłowski, 1962 (Erpobdellidae), and *Mimobdella japonica* Blanchard, 1897 (Salifidae), were included.

**Table 1.** Samples used for the phylogenetic analyses. The information on voucher, collection locality, and GenBank accession numbers are indicated.

**Species**	**Voucher**	**Locality**	**COI**	**12S**
*Orobdella esulcata*	KUZ Z29 Holotype	Kumamoto, Japan (32°48.60'N, 130°38.48'E)	AB679664	AB679665
*Orobdella esulcata*	KUZ Z170	Ikinoshima Isl., Japan (33°44.47'N, 129°42.25'E)	AB679666	AB679667
*Orobdella dolichopharynx*	KUZ Z120 Holotype	Amamioshima Isl., Japan (28°17.18'N, 129°18.93'E)	AB679680	AB679681
*Orobdella dolichopahrynx*	KUZ Z122	Kinsakubaru, Amamioshima Isl., Japan	AB679682	AB679683
*Orobdella ijimai*	KUZ Z110 Topotype	Tochigi, Japan (36°46.98'N, 139°34.93'E)	AB679672	AB679673
*Orobdella ijimai*	KUZ Z188	Nagano, Japan (36°12.44'N, 138°37.74'E)	AB679674	AB679675
*Orobdella kawakatsuorum*	KUZ Z148	Toyotomi, Hokkaido, Japan (45°13.22'N, 141°41.07'E)	AB679692	AB679693
*Orobdella kawakatsuorum*	KUZ Z150	Richirito Isl., Hokkaido, Japan (45°11.99'N, 141°14.26'E)	AB679694	AB679695
*Orobdella kawakatsuorum*	KUZ Z152	Shari, Hokkaido, Japan (44°06.09'N, 145°06.09'E)	AB679696	AB679697
*Orobdella kawakatsuorum*	KUZ Z153	Ashoro, Hokkaido, Japan (43°23.70'N, 143°59.23'E)	AB679698	AB679699
*Orobdella kawakatsuorum*	KUZ Z154	Kamikawa, Hokkaido, Japan (43°43.55'N, 142°57.53'E)	AB679700	AB679701
*Orobdella kawakatsuorum*	KUZ Z159	Kyowa, Hokkaido, Japan (42°56.17'N, 140°35.57'E)	AB679702	AB679703
*Orobdella kawakatsuorum*	KUZ Z167	Sapporo, Hokkaido, Japan (43°03.15'N, 141°18.71'E)	AB679704	AB679705
*Orobdella koikei*	KUZ Z145	Hiratori, Hokkaido, Japan (42°40.82'N, 142°25.44'E)	AB679684	AB679685
*Orobdella koikei*	KUZ Z146	Shinhidaka, Hokkaido, Japan (42°42.86'N, 142°38.30'E)	AB679686	AB679687
*Orobdella koikei*	KUZ Z156 Holotype	Kamikawa, Hokkaido, Japan (43°43.36'N, 142°56.85'E)	AB679688	AB679689
*Orobdella koikei*	KUZ Z158	Mashike, Hokkaido, Japan (43°46.23'N, 141°30.63'E)	AB679690	AB679691
*Orobdella octonaria*	KUZ Z177	Tokyo, Japan (35°42.94'N, 139°12.20'E)	AB679706	AB679707
*Orobdella octonaria*	KUZ Z181 Topotype	Kanagawa, Japan (35°14.06'N, 139°04.21'E)	AB679708	AB679709
*Orobdella shimadae*	KUZ Z128 Holotype	Okinawajima Isl., Japan (26°49.08'N, 128°16.90'E)	AB679676	AB679677
*Orobdella shimadae*	KUZ Z138	Okinawajima Isl., Japan (26°40.20'N, 128°11.20'E)	AB679678	AB679679
*Orobdella tsushimensis*	KUZ Z133	Tsushimajima Isl., Japan (34°34.66'N, 129°22.49'E)	AB679660	AB679661
*Orobdella tsushimensis*	KUZ Z134 Holotype	Tsushimajima Isl., Japan (34°15.29'N, 129°17.28'E)	AB679662	AB679663
*Orobdella whitmani*	KUZ Z45 Topotype	Gifu, Japan (35°25.65'N, 136°46.91'E)	AB679668	AB679669
*Orobdella whitmani*	KUZ Z191	Shiga, Japan (35°39.63'N, 136°11.30'E)	AB679670	AB679671
*Erpobdella japonica*	KUZ Z178	Nagano, Japan (36°12.43'N, 138°36.93'E)	AB679654	AB679655
*Gastrostomobdella monticola*	UNIMAS/A03/BH01/10	Kuching, Malaysia	AB679656	AB679657
*Mimobdella japonica*	KUZ Z179	Amamioshima Isl., Japan (28°26.53'N, 129°33.60'E)	AB679658	AB679659

Voucher specimens used in this study have been deposited in the National Museum of Nature and Science, Tokyo (NSMT), the Universiti Malaysia Sarawak (UNIMAS), and the Zoological Collection of Kyoto University (KUZ).

### PCR and DNA sequencing

Genomic DNA was extracted from botryoidal tissues preserved in 99% ethanol using a modification of the method in Okamoto et al. (2006). After digestion of botryoidal tissues with proteinase K (100 μg/ml) at 37°C for eight–ten hours, DNA was extracted two times with phenol and one time with 25:24:1 phenol/chloroform/isoamyl-alcohol, and precipitated in two volumes of 99% ethanol with one-tenth volume of 3.0 M sodium acetate (pH 5.2). Precipitated samples were dried and stored in TE buffer (10 mM Tris-HCl and 1 mM EDTA [pH 8.0]). Primer sets used in this study are listed in Table 2: for COI, LCO1490 and HCO2198 (Folmer et al. 1994), and LCO-in and HCO-out; for tRNA^Cys^, tRNA^Met^, 12S, tRNA^Val^ and 16S (abbreviated 12S), 12SA-out and 12SB-in, and 12SA-in and 12SB-out. All amplification reactions were performed in a GeneAmp PCR System 2700 (Applied Biosystems) or a MyCycler (Bi-Rad Laboratories) using an Ex *Taq* Polymerase Kit (Takara Bio Inc.). Reaction mixtures were heated to 94°C for 5 min, followed by 35 cycles of 94°C (10 s), 42.5°C (20 s), and 72°C (1 min 13 s for COI, and 1 min for 12S) and a final extension at 72°C for 6 min. The amplified DNA fragments were purified using polyethylene glycol (20% PEG 6000) precipitation.

**Table 2.** PCR and cycle sequencing (CS) primers used in this study.

**Gene**	**Primer name**	**Reaction**	**Primer sequence (5’→ 3’)**	**Source**
COI				
1	LCO1490	PRC & CS	GGTCAACAAATCATAAAGATATTGG	Folmer et al. (1994)
	HCO2198	CS	TAAACTTCAGGGTGACCAAAAAATCA	Folmer et al. (1994)
2	LCO-in	CS	TCCAGAACGTATTCCATTATTTG	This study
	HCO-out	PCR & CS	TCTGGGTAGTCAGAATATCG	This study
tRNA^Cys^, tRNA^Met^, 12S rDNA, tRNA^Val^ and 16S rDNA				
1	12SA-out	PCR & CS	TTGATGAACAACATTAAATTGC	This study
	12SB-in	CS	TAAGCTGCACTTTGACCTGA	This study
2	12SA-in	CS	AATTAAAACAAGGATTAGATACCC	This study
	12SB-out	PCR & CS	AACCCATAATGCAAAAGGTAC	This study

All samples were sequenced in both directions. Sequencing reactions were performed using a BigDye Terminator v3.1 Cycle Sequencing Kit (Applied Biosystems). Each sequencing reaction mixture was incubated at 96°C for 2 min, followed by 40 cycles of 96°C (10 s), 50°C (5 s), and 60°C (45 s for COI, and 40 s for 12S). The products were collected by ethanol precipitation and sequenced on an ABI 3130xl Genetic Analyzer (Applied Biosystems). Obtained sequences were edited using DNA BASER (Heracle Biosoft S.R.L.). These sequence data were deposited in GenBank.

### Phylogenetic analyses

COI sequences were aligned by eye since there were no indels. Mitochondrial 12S sequences were aligned using MAFFT X-INS-i (Hofacker et al. 2002, Katoh and Toh 2008, McCaskill 1990, Tabei et al. 2008) taking into account RNA secondary structure information, and then refined with GBLOCKS (Castresana 2000). The length of aligned sequences of COI was 1266 bp, and that of 12S was 718 bp. Prior to phylogenetic analyses, transition/transversion (ti/tv) rate ratios for each gene sequence was calculated using MEGA5 (Tamura et al. 2011) to test for saturation in base substitutions. It was confirmed that COI and 12S did not show any signs of saturation (ti/tv rate ration of COI was 1.02, and that of 12S was 1.07). Therefore, the concatenated sequences yielded a total of 1984 bp positions.

Phylogenetic tree were constructed using maximum likelihood (ML) and Bayesian inference (BI). Pairwise comparisons of Kimura-2 parameter (K2p) distance (Kimura 1980) were also calculated using MEGA5. ML phylogenies were calculated using TREEFINDER v October 2008 (Jobb et al. 2004) with the tool package PHYLOGEARS v 2.0 (Tanabe 2008), and then non-parametric bootstrapping (Felsenstein 1985) was conducted with 500 replicates. The best-fit models for each partition were selected using the Akaike Information Criterion (Akaike 1974) by using KAKUSAN4 (Tanabe 2011). For the 1st position of COI, the Tamura-Nei model (TN93) with gamma distribution (+G) and proportion of invariant sites (+I) was selected. The transversion model (TVM)+I was selected for the 2nd position, the transition model (TIM)+G for the 3rd position of COI, and the general time reversal model (GTR)+G for 12S. BI and Bayesian posterior probabilities (BPPs) were estimated using the MPI version of MRBAYES v 3.1.2 (Altekar et al. 2004, Huelsenbeck et al. 2001, Ronquist and Huelsenbeck 2003). The best-fit models for each partition were identified using the Bayesian Information Criterion (Schwarz 1978) also by using KAKUSAN4: for COI 1st position, GTR+G+I; the Felsenstein 1981 model (F81)+I for COI 2nd position; the Hasegawa-Kishino-Yano model (HKY85)+G for COI 3rd position; and GTR+G for 12S. Two independent runs for four Markov chains were conducted for 1.5 million generations and the tree was sampled every 100 generations. Based on checking the parameter estimates and convergence using TRACER v 1.5 (Rambaut and Drummond 2009), the first 5,001 trees were discarded.

The nodes with bootstrap value (BS) higher than 70% were regarded as sufficiently resolved (Hillis and Bull 1993). Nodes with BPP higher than 95% were considered statistically significant (Leaché and Reeder 2002).

## Results

### Taxonomy

**Genus *Orobdella* Oka, 1895**

#### Orobdella koikei sp. n.

urn:lsid:zoobank.org:act:7DBE6F21-E4C3-4CBF-9469-2B13121F56D4

http://species-id.net/wiki/Orobdella_koikei

**Figure 2.**
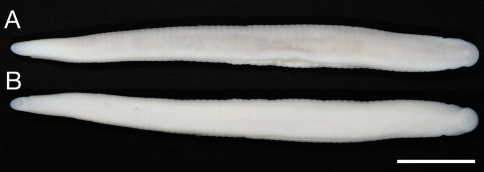
*Orobdella koikei* sp. n., holotype, KUZ Z156 **A** Dorsal and **B** ventral views. Scale bar, 5 mm.

Figs 2 [Fig F3] [Fig F4] –5

##### Diagnosis.

 In life, dorsal surface brown. Somites III and IV uniannulate, somites VIII–XXIV quadrannulate, somites XXV and XXVI triannulate. Pharynx reaching to XIV. Gastropore conspicuous at XIII a1 (slightly posterior to middle of annulus). Gastroporal duct, tubular, but bulbous at junction with gastropore and at junction with crop. Male gonopore at XI b6, female gonopore at XIII a1 (slightly posterior to middle of annulus), gonopores separated by 1/2 + 4 + 1/2. Paired epididymides in XVI/XVII–XVII a2 to XIX a2/b5. Atrial cornua ovate.

##### Type materials.

 KUZ Z156, **holotype**, dissected, collected from under a rock along a mountain trail at Sounkyo, Kamikawa, Hokkaido, Japan (43°43.36'N, 142°56.85'E; Alt. 712 m), by Naoki Koike on 17 August, 2010.

Five **paratypes** collected from Hokkaido, Japan. Two specimens from the type locality (43°43.36'N, 142°56.85'E; Alt. 712 m): KUZ Z157, dissected, by Naoki Koike on 17 August, 2010, and KUZ Z186, by TN on 19 September, 2011. KUZ Z145, dissected, from Hiratori (42°40.82'N, 142°25.44'E; Alt. 220 m), by Naoki Koike on 2 August, 2010. KUZ Z146, dissected, from Mt. Pisenaiyama, Shinhidaka (42°42.86'N, 142°38.30'E; Alt. 981 m), by Naoki Koike on 3 August, 2010. KUZ Z158, dissected, from Mt. Shokanbetsudake, Mashike (43°46.23'N, 141°30.63'E; Alt. 288 m), by Naoki Koike on 18 August, 2010.

##### Etymology.

 The specific name is a noun in the genitive case formed directly from the name of Mr Naoki Koike, who collected many valuable specimens of *Orobdella* leeches from Hokkaido.

##### Description of holotype.

**Figure 3. F3:**
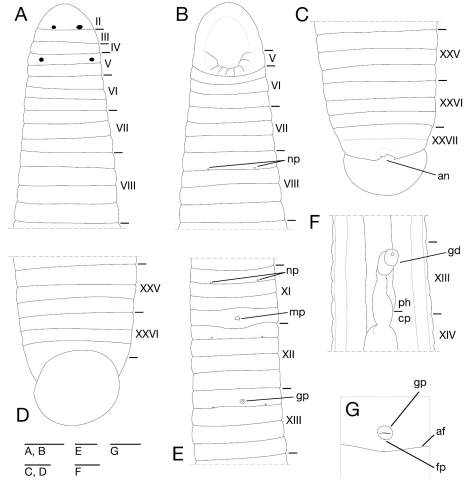
*Orobdella koikei* sp. n., holotype, KUZ Z156 **A** Dorsal view of somites I–VIII **B** ventral view of somites I–VIII **C** dorsal view of somites XXV–XXVII and caudal sucker **D** ventral view of somites XXV–XXVII and caudal sucker **E** ventral view of somites XI–XIII **F** ventral view of gastroporal duct; and **G** ventral view of gastropore and female gonopore. Scale bars, 0.5 mm (**A–F**) and 0.25 mm (**G**). Abbreviations: af, annular furrow; an, anus; cp, crop; fp, female gonopore; gd, gastroporal duct; gp, gastropore; mp, male gonopore; np, nephridiopore; and ph, pharynx.

 Body firm, muscular, elongated, gaining regularly in width in caudal direction, dorso-ventral depressed, sides nearly parallel from mid length to point just anterior to caudal sucker, BL 30.5 mm, BW 2.5 mm (Fig. 2). Caudal sucker ventral, oval, its diameter smaller than BW (Figs 2B, 3D). In life, dorsal surface brown, ventral surface grayish white. Color faded in preservative, without any dark lines (Fig. 2)

Somite I completely merged with prostomium (Fig. 3A). Somites II–IV uniannulate (Fig. 3A). Somite V biannulate, (a1+a2) = a3 (Fig. 3A), V a3 forming posterior margin of oral sucker (Fig. 3B). Somites VI and VII triannulate (Fig. 3A–B). Somites VIII–XXIV quadrannulate, a1 = a2 = b5 = b6 (Fig. 3A–B, E). Somites XXV and XXVI triannulate (Fig. 3C–D), XXVI a3 being last complete annulus on venter (Fig. 3D). Somite XXVII uniannulate with one slight furrow on dorsal; anus behind it with no post-anal annulus (Fig. 3C).

**Figure 4. F4:**
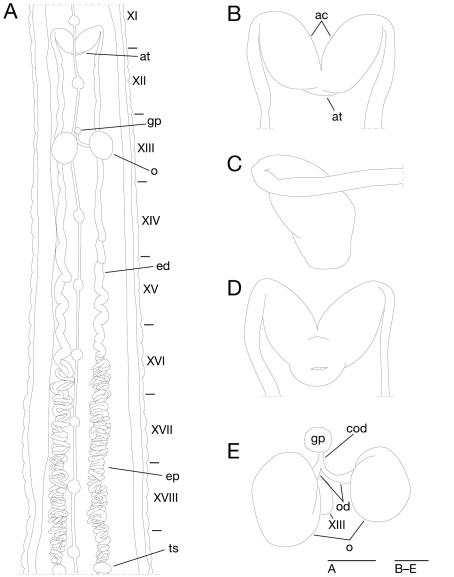
*Orobdella koikei* sp. n., holotype, KUZ Z156 **A** Dorsal view of reproductive system including ventral nervous system **B** dorsal view of male atrium **C** lateral view of male atrium **D** ventral view of male atrium; and **E** dorsal view of female reproductive system including position of ganglion XIII. Scale bars, 1mm (**A**) and 0.25 mm (**B–E**). Abbreviations: ac, atrial cornu; at, atrium; cod, common oviduct; ed, ejaculatory duct; ep, epididymis; gp, gastropore; o, ovisac; od, oviduct; and ts, testisac.

Anterior ganglionic mass in VI a2 and a3. Ganglion VII in a2. Ganglion VIII in a2 and b5. Ganglion IX in a2. Ganglia X–XII in a2 and b5 of each somite (Fig. 4A). Ganglion XIII in b5 (Fig. 4A). Ganglia XIV and XV in a2 and b5 of each somite (Fig. 4A). Ganglia XVI–XXI in a2 of each somite (Fig. 4A). Ganglia XXII–XXIV in a1 and a2 of each somite. Ganglia XXV and XXVI in a1 of each somite. Posterior ganglionic mass in XXVII a2 and a3.

Eyes two pairs, first pair dorsally on posterior margin of II, second pair dorsolaterally on middle of V (a1 + a2) (Fig. 3A). Nephridiopores in 17 pairs, ventrally at posterior margin of a1 of each somite of VIII–XXIV (Fig. 3A, E). Papillae numerous, minute, hardly visible, one row on every annulus.

Pharynx agnathous, euthylaematous, reaching to XIII/XIV (Fig. 3F). Crop tubular, acaecate, in XIII/XIV to XIX b5/b6. Gastropore conspicuous, ventral, located slightly posterior to middle of XIII a2 (Fig. 3E, G). Gastroporal duct, winding and bulbous at junction with gastropore, tubular but bulbous at junction with crop, joining with crop in XIV a1 (Fig. 3F). Intestine tubular, acaecate, in XIX b5/b6 to XXIII a2. Rectum, tubular, thin-walled.

Male gonopore at middle of XI b6 (Fig. 3E). Female gonopore located slightly posterior to middle of XIII a2, inconspicuous, located behind gastropore (Fig. 3G). Gonopores separated by 1/2 + 4 + 1/2 annuli (Fig. 3E). Testisacs multiple, one or two testisacs on each side in each annulus, in XIX a2/b5 to XXIV a1 (Fig. 4A). Paired epididymides in XVI a2/b5 to XIX a2/b5 (Fig. 4A). Ejaculatory bulbs absent. Ejaculatory ducts in XI a2/b5 to XVI a2/b5, loosely coiled, each winding from each junction with epididymis, narrowing at junction with atrial cornu, then turning sharply inward toward atrial cornu without pre-atrial loop (Fig. 4A–D). Pair of atrial cornua in XI b5 and b6, muscular, ovate (Fig. 4B). Atrium short, muscular, globular in XI b6 (Fig. 4B–D). Penis sheath and penis absent. Ovisacs one pair, thin-walled, globular, in XIII a2 and b5 (Fig. 4A, E). Oviducts thin-walled, left oviduct crossing ventrally beneath nerve cord, both oviducts converging into common oviduct in XIII a2 (Fig. 4A, E). Common oviduct thin-walled, short, directly ascending to female gonopore (Fig. 4E).

##### Variation.

**Figure 5.**
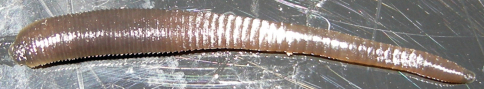
*Orobdella koikei* sp. n., paratype, KUZ Z186, taken of live animal, dorsal view.

 In life, color generally same as holotype (Fig. 5). Somite III with slight furrow on dorsal (KUZ Z146). Somite IV with slight furrow on dorsal (KUZ Z158), or biannulate (KUZ Z146). Somite XXVI incomplete triannulate. Pharynx reaching to XIII b5/b6–XIV a1. Crop reaching to XIX b5–XX a1. Gastropore at middle of XIII. Gastroporal duct simple tubular (KUZ Z145 and Z146). Intestine reaching to XXIII a1–XXIV a2. Female gonopore at middle of XIII. Testisacs in XVIII a1–XIX a2/b5 to XXIII a2/b5. Epididymides in XVI/XVII–XVII a2 to XIX a2/b5. Right or left oviduct crossing ventrally beneath nerve cord.

##### Distribution.

 Known in mountainous regions of the central part of Hokkaido, Japan (Fig. 1).

##### Remarks.

 The specimens examined in this study consist of small individuals. However, testisacs and ovisacs of the holotype, of which BL is 30.5 mm, are developed. In immature *Orobdella* specimens, testiscas are usually undeveloped, and hardly detected (Nakano pers. obs.). Therefore, there is a possibility that the holotype of this species is a mature leech.

*Orobdella koikei* is syntopic with *Orobdella kawakatsuorum* at Sounkyo (Locality No. 2 in Fig. 1). Specimens of *Orobdella koikei* collected from Sounkyo were clearly distinguished from those of *Orobdella kawakatsuorum* in Sounkyo by the number of annuli between the gonopores, annulation of XXV, morphology of the gastroporal duct and male atrium, and the length of epididymides. Therefore, *Orobdella koikei* can be treated as a distinct new species from Hokkaido.

#### Orobdella kawakatsuorum

Richardson, 1975

http://species-id.net/wiki/Orobdella_kawakatsuorum

**Figure 6.**
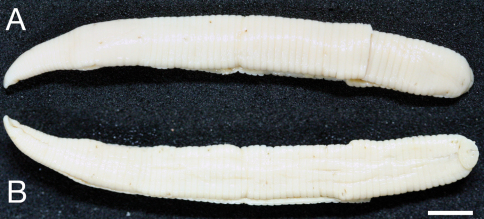
*Orobdella kawakatsuorum* Richardson, 1975, holotype, NSMT-An 53 **A** Dorsal and **B** ventral views. Scale bar, 5 mm.

Figs 6 [Fig F7] [Fig F8] [Fig F9] [Fig F10] –11

Orobdella kawakatsuorum Richardson, 1975: 42–51, figs 1, 2; Sawyer 1986: 680, 747.

##### Diagnosis.

 In life, dorsal surface grayish blue. Somites III and IV biannulate, somites VIII–XXV quadrannulate, somite XXVI triannulate, clitellum from X b5 to XIII a2. Pharynx reaching to XIV. Gastropore conspicuous in furrow of XIII a1/a2. Gastroporal duct, simple tubular. Male gonopore in furrow of XI b5/b6, female gonopore in furrow of XIII a1/a2, gonopores separated by 6 annuli. Paired epididymides in XVI a2/b5–XVII b5 to XVI b5–XVII b6. Atrial cornua, coniform, undeveloped.

##### Material examined.

 NSMT-An 53, **holotype**, dissected by Richardson, LR, collected from a home garden of Professor Masaharu Kawakatsu, Sapporo, Hokkaido, Japan, by Tetsuya Kawakatsu and Miyuki Kawakatsu on 1 June, 1974.

Additional materials. 22 specimens collected from Hokkaido, Japan. Six specimens collected from Maruyama–koen Park, Chuo–ku, Sapporo: KUZ Z24, dissected, and Z140 (43°03.12'N, 141°18.53'E; Alt. 50 m) by Naoyuki Nakahama on 14 June, 2009; KUZ Z166, Z167, dissected (43°03.15'N, 141°18.71'E; Alt. 34 m), Z168 and Z169, dissected (43°03.15'N, 141°18.61'E; Alt. 29m), by TN on 5 October, 2010. Six specimens from Sounkyo, Kamikawa: KUZ Z154, dissected, and Z155 (43°43.55'N, 142°57.53'E; Alt. 705 m), by Naoki Koike on 16 August, 2010; KUZ Z183 (43°43.45'N, 142°56.86'E; Alt. 674 m), Z184 and Z185 (43°43.39'N, 142°56.88'E; Alt. 678 m), and Z187 (43°43.29'N, 142°56.87'E; Alt. 758 m), by TN on 19 September, 2011. Two specimens from Mt. Asahidake, Higashikawa by Naoyuki Nakahama: KUZ Z141, dissected (43°38.82'N, 142°47.73'E; Alt. 1090 m) on 16 June, 2009, and KUZ Z142 (43°39.12'N, 142°48.10'E; Alt. 1120 m) on 17 June, 2009. Two specimens from near the Kabutonuma Pond, Toyotomi (45°13.22'N, 141°41.07'E; Alt. 16 m), by Naoki Koike on 6 August, 2010: KUZ Z147 and Z148, dissected. Two specimens from Mt. Rishirizan, Rishirifuji (Rishirito Island) (45°11.99'N, 141°14.26'E; Alt. 914 m), by Naoki Koike on 8 August, 2010: KUZ Z149 and Z150, dissected. KUZ Z143, dissected, from Nukabira, Kamishihoro (43°22.05'N, 143°11.62'E; Alt. 490 m), by Naoyuki Nakahama on 18 June, 2009. KUZ Z152, dissected, from Mt. Rausudake, Shari (44°06.09'N, 145°06.09'E; Alt. 630 m), by Naoki Koike on 13 August, 2010. KUZ Z153, dissected, from Mt. Meakandake, Ashoro (43°23.70'N, 143°59.23'E; Alt. 755 m), by Naoki Koike on 15 August, 2010. KUZ Z159, dissected, from near the Shinsennuma Pond, Kyowa (42°56.17'N, 140°35.57'E; Alt. 781 m), by Naoki Koike on 19 August, 2010.

##### Emended description.

**Figure 7. F7:**
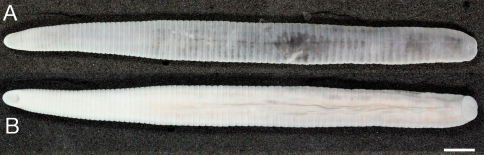
*Orobdella kawakatsuorum* Richardson, 1975, collected from near the type locality, KUZ Z167 **A** Dorsal and **B** ventral views. Scale bar, 5 mm

**Figure 8. F8:**
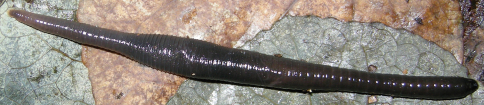
*Orobdella kawakatsuorum* Richardson, 1975, collected from near the type locality, KUZ Z167, taken of live animal, dorsal view.

**Figure 9. F9:**
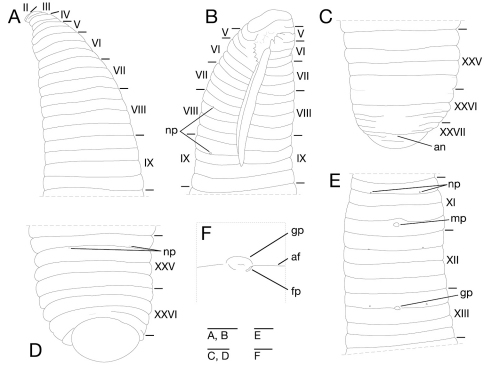
*Orobdella kawakatsuorum* Richardson, 1975, holotype, NSMT-An 53 **A** Dorsal view of somites I–IX **B** ventral view of somites I–IX **C** dorsal view of somites XXV–XXVII and caudal sucker **D** ventral view of somites XXV–XXVII and caudal sucker **E** ventral view of somites XI–XIII; and **F** ventral view of gastropore and female gonopore. Scale bars, 1 mm (**A–E**) and 0.25 mm (**F**). Abbreviations, see Fig. 3.

**Figure 10. F10:**
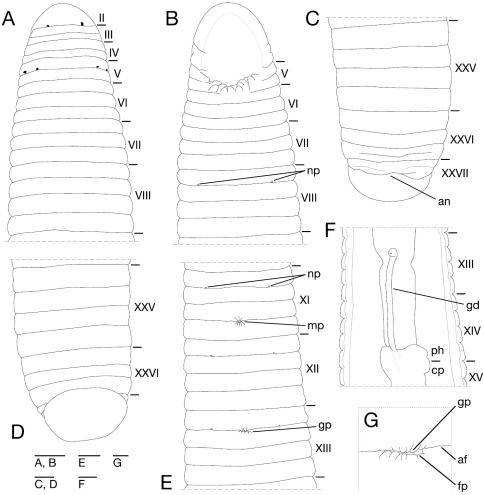
*Orobdella kawakatsuorum* Richardson, 1975, collected from near the type locality, KUZ Z167 **A** Dorsal view of somites I–VIII **B** ventral view of somites I–VIII **C** dorsal view of somites XXV–XXVII and caudal sucker **D** ventral view of somites XXV–XXVII and caudal sucker **E** ventral view of somites XI–XIII **F** ventral view of gastroporal duct; and **G** ventral view of gastropore and female gonopore. Scale bars, 1 mm (**A–F**) and 0.25 mm (**G**). Abbreviations, see Fig. 3.

 Body firm, muscular, elongated, gaining regularly in width in caudal direction, dorso-ventral depressed, sides nearly parallel from mid length to point just anterior of caudal sucker (Figs 6, 7), maximu BL 111.64 (KUZ Z142), maximun BW 8.19 (KUZ Z154). Caudal sucker ventral, ova, its diameter smaller than BW (Figs 6, 7, 9D, 10D). In life, dorsal surface grayish blue, ventral surface bluish white (Fig. 8). Color faded in preservative, without any dark lines (Figs 6, 7).

Somite I completely merged with prostomium (Fig. 10A). Somite II uniannulate (Figs 9A, 10A). Somite III uniannulate in small specimens, biannulate in large specimens (Figs 9A, 10A). Somite IV generally biannulate (Figs 9A, 10A), but uniannulate in a few small specimens. Somite V biannulate, (a1 + a2) = a3, V a3 forming posterior margin of oral sucker (Figs 9A–B, 10A–B). Somites VI and VII triannulate, a1 = a2 = a3 (Figs 9A–B, 10A–B). Somites VIII–XXV quadrannulate, a1 = a2 = b5 = b6 (Figs 9A–E, 10A–E); X b5 being first annulus of clitellum, XIII a2 being last annulus of clitellum. Somite XXVI triannulate, a1 = a2 = a3, a3 being last complete annulus on venter (Figs 9C–D, 10C–D); a3 with furrow on dorsal in large specimens (Fig. 10C). Somite XXVII uniannulate, or biannulate; anus behind it with no post-anal annulus (Fig. 9C, 10C).

**Figure 11.**
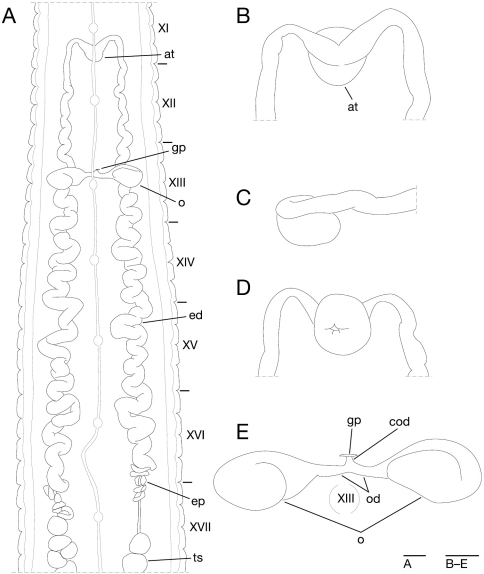
*Orobdella kawakatsuorum* Richardson, 1975, collected from near the type locality, KUZ Z167 **A** Dorsal view of reproductive system including ventral nervous system **B** dorsal view of male atrium **C** lateral view of male atrium **D** ventral view of male atrium; and **E** dorsal view of female reproductive system including position of ganglion XIII. Scale bars, 1mm (**A**) and 0.5 mm (**B–E**). Abbreviations, see Fig. 4.

Anterior ganglionic mass in VI a2– VII a1. Ganglion VII in a2, a2 and a3, or a3. Ganglia VIII–XV mainly in a2 of each somite, but also a2 and b5, or b5 in several specimens (Fig. 11A). Ganglion XVI in a2 (Fig. 11A). Ganglia XVII–XXIV generally in a2 of each somite (Fig. 11A), but rarely in a1 and a2, or a2 and b5. Ganglion XXV generally in a1, but also a1 and a2, or a2 in several specimens. Ganglion XXVI in a1, XXV b6, XXV b6 and XXVI a1, or XXVI a2. Posterior ganglionic mass in XXVI a1–a3.

Eyes three pairs, first pair dorsally on posterior margin of II, second and third pairs dorsolaterally on posterior margin of V (a1 + a2) (Fig. 10A); eyes one pair in several large specimens, dorsally on posterior margin of II. Nephridiopores in 17 pairs, ventrally at posterior margin of a1 of each somite of VIII–XXIV (Fig. 10B, E); rarely in 18 pairs at a1 of each somite of VIII–XXV (NSMT-An 53, KUZ Z24, Z143, and Z155) (Fig. 9B, D–E). Papillae numerous, minute, hardly visible, one row on every annulus.

Pharynx agnathous, euthylaematous, reaching to XIV a2–XIV/XV (Fig. 10F). Crop tubular, acaecate, in XIVa2–XIV/XV to XX b5/b6–XXI a1/a2. Gastropore conspicuous, ventral, generally in furrow of XIII a1/a2 (Figs 9E–F, 10E, G). Gastroporal duct, simple tubular, thin-walled, joining with crop in XIV a2–XIV/XV (Fig. 10F). Intestine tubular, acaecate, in XIV a2–XIV/XV to XXIII b5–XIV b5/b6. Rectum, tubular, thin-walled.

Male gonopore generally in furrow of XI b5/6, or at anterior part of XI b6 (Figs 9E, 10E). Female gonopore in furrow of XIII a1/a2, located behind gastropore (Figs 9F, 10G). Gonopores separated by six annuli (Figs 9E, 10E). Testisacs multiple, two or three testisacs on each side in each annulus, in XVI b5–XVII b6 to XXIII a1–XXV b6 (Fig. 11A). Paired epididymides in XVI a2/b5–XVII b5 to XVI b5–XVII b6 (Fig. 11A). Ejaculatory bulbs absent. Ejaculatory ducts in XI b5 to XVI a2/b5–XVII b5, loosely coiled, each winding from each junction with epididymis, narrowing at junction with atrial cornu, then turning inward toward atrial cornu without pre-atrial loop (Fig. 11A–D). Pair of atrial cornua in XI b5 and b6, undeveloped, coniform (Fig. 11B). Atrium short, muscular, globular in XI b5 and b6 (Fig. 11B–D). Ovisacs one pair, thin-walled, globular, in XIII a2 and b5 (Fig. 11A, E). Oviducts thin-walled, right or left oviduct crossing ventrally beneath nerve cord, both oviducts converging into common oviduct in XIII a2 (Fig. 11A, E). Common oviduct thin-walled, short, directly ascending to female gonopore (Fig. 11E).

##### Distribution.

 Known in mountainous regions of Hokkaido, Japan (Fig. 1).

##### Remarks.

Richardson (1975) described that a gastropore of the holotype opened at the middle of XIII a1, and the female gonopore in the furrow of XIII a1/a2. However, both the gastropore and the female gonopore of *Orobdella kawakatsuorum* are in the furrow of XIII a1/a2 on the basis of examination of the holotype and newly collected specimens. A gastropore of this species is coincident with a female gonopore. Richardson also noted that a pair of nephririopores opened in XXV (XXIV in his paper). But it is rare for *Orobdella kawakatsuorum* to possess 18 pairs of nephridiopores.

### Phylogenetic relationships

**Figure 12.**
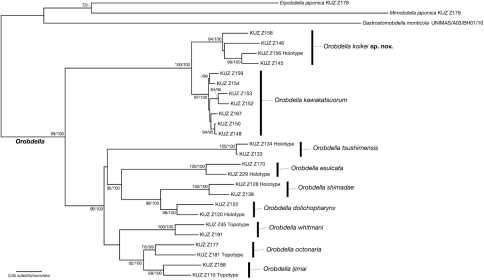
The ML tree of 1984 bp of mitochondrial COI, tRNA^Cys^, tRNA^Met^, 12S rDNA, tRNA^Val^ and 16S rDNA. The numbers associated with the nodes represent the bootstrap values for ML (BS)/ and Baysian posterior probabilities (BPPs). BS higher than 70% and/or BPP higher than 95% are indicated.

The ML tree with ln *L* = -12757.40 (Fig. 12) was nearly identical to the obtained BI tree (not shown). Monophyly of the genus *Orobdella* was well supported (BS = 99%, BPP = 100%). Two *Orobdella* species from Hokkaido, *Orobdella koikei* and *Orobdella kawakatsuorum*, formed a monophyletic group (BS = 100%, BPP = 100%). This clade was a sister taxon of the other *Orobdella* species. Monophyly of *Orobdella koikei* and *Orobdella kawakatsuorum* was well supported (*Orobdella koikei*: BS = 94%, BPP = 100%; *Orobdella kawakatsuorum*: BS = 97%, BPP = 100%).

The COI sequence divergence between *Orobdella koikei* and *Orobdella kawakatsuorum* was between 8.1–9.9% (mean = 9.0%). Intraspecific variation of COI sequences ranged between 4.8–8.1% (mean = 7.1%) in *Orobdella koikei*, and 0.30–4.9% (mean = 3.7%) in *Orobdella kawakatsuorum*. Sequence divergence of 12S between these two species was between 3.3–5.5% (mean = 4.3%). It was between 2.8–4.8% (mean = 3.9%) within *Orobdella koikei*, and 0–1.1% (mean = 0.71%) within *Orobdella kawakatsuorum*. Interspecific genetic distance of COI between *Orobdella koikei* and *Orobdella kawakatsuorum* had a significantly higher mean divergence as compared to the intraspecific variation of *Orobdella koikei* (*t*-test with unequal variance, *p* = 0.0096), but that of 12S did not have a significantly higher mean divergence (*t*-test with unequal variance, *p* = 0.22).

Monophyly of seven *Orobdella* species distributed in areas south of Hokkaido was recovered (BS = 95%, BPP = 100%). Monophyly of *Orobdella esulcata* + *Orobdella shimadae* + *Orobdella dolichopharynx* received strong support (BS = 95%, BPP = 100%), and that of *Orobdella jimai* + *Orobdella octonaria* was also recovered (BS = 92%, BPP = 100%).

## Discussion

*Orobdella koikei* differs from the four other quadrannulate species of the genus, *Orobdella esulcata*, *Orobdella kawakatsuorum*, *Orobdella tsushimensis*, and *Orobdella whitmani*, in the following combination of characteristics (Table 3): 1) dorsal surface brown ; 2) IV uniannulate; 3) male gonopore at XI b6; 4) gastropore nad female gonopore at XIII a1; 5) gonopores separated by 1/2 + 4 + 1/2 annuli; 6) XXV triannulate; 7) gastroporal duct, tubular, but bulbous at junctions with gastropore and crop; 8) epididymides in XVII to XIX; and 9) atrial cornua ovate. *Orobdella koikei* is easily distinguished from *Orobdella dolichopharynx* Nakano, 2011, *Orobdella ijimai* Oka, 1895, *Orobdella shimadae* Nakano, 2011, and *Orobdella octonaria* Oka, 1895, in having mid-body somites that are quadrannulate; they are sexannulate in *Orobdella dolichopharynx*, *Orobdella ijimai*, and *Orobdella shimadae*, and octannulate in *Orobdella octonaria*.

**Table 3.** Comparisons of morphological characters between *Orobdella koikei* sp. n. and four quadrannulate congeneric species.

**Character**	***Orobdella koikei* sp. n.**	***Orobdella esulcata***	***Orobdella kawakatsuorum***	***Orobdella tsushimensis***	***Orobdella whitmani***
Color	brownish	bluish	bluish	yellowish	yellowish
Annulation of IV	uniannulate	uniannulate	biannulate	unianulate	uni- or biannulate
Number of annuli between gonopores	1/2 + 4 + 1/2	2/3 + 4 + 1/3	6	1/2 + 5	1/2 + 4 + 1/2
Annulation of XXV	triannulate	quadrannulate	quadrannulate	quadrannulate	quadrannulate
Gastroporal duct	tubular, but bulbous at junctions with gastropore and crop	tubular, but bulbous at junction with gastropore	simple tubular	bottle-shaped	bulbiform
Epididymides	XVII to XIX	XVI to XX	XVI to XVII	XVI to XIX	XVI to XVIII
Atrial cornua	ovate	ovate	undeveloped	coniform	ovate

The phylogenetic tree showed that the clade, which includes *Orobdella* species in Hokkaido, was a sister taxon of the other *Orobdella* species. This result is also recovered by the other phylogenetic analyses based on nucleic 18S and 28S sequences (Nakano et al. in press). According to the phylogenetic analyses, several characteristics are considered to have evolved in parallel. Each of *Orobdella kawakatsuorum* and *Orobdella esulcata* possess a tubular gastroporal duct (Nakano 2010). However, these two species are phylogenetically distant. The mid-body somite annulation of *Orobdella* leeches does not indicate phylogenetic relationships either. In the genus *Orobdella*, it is clear that the quadrannulate mid-body somite is a plesiomorphic character. Sexannulate mid-body somite, which *Orobdella dolichopharynx*, *Orobdella ijimai*, and *Orobdella shimadae* possess, evolved in parallel. *Orobdella kawakatsuorum*, and two species from Ryukyu Archipelago, Japan, *Orobdella dolichopharynx* and *Orobdella shimadae*, possess rudimentary male atrial cornua (Nakano 2011b). However, undeveloped male atrial cornua do not indicate any phylogenetic relationships between *Orobdella kawakatsuorum* and Ryukyu *Orobdella* species. These characters are not useful for estimating phylogenetic relationships in the genus *Orobdella*, although they are suitable for the species level classification.

Species delimitation in leeches based on genetic analyses, especially using COI DNA-barcode locus, has been discussed in many papers (see DeSalle et al. 2005 for review). The average sequence divergence of COI between *Orobdella koikei* and *Orobdella kawakatsuorum* was 9.0%, and that of 12S was 4.3%. Interspecific genetic divergence of COI between these two species showed a significantly higher value than that of the intraspecific variation, although the intraspecific genetic divergence of 12S sequences in *Orobdella koikei* (2.8–4.8%) was overlapped to a large extent with the interspecific divergence of 12S (3.3–5.5%). Thus, only the genetic distance of COI (mean = 9.0%) can be used as an indicator for deciding whether leeches are distinct species or not in the genus *Orobdella*, since *Orobdella koikei* and *Orobdella kawakatsuorum* are distributed syntopically at Sounkyo (Fig. 1).

Gilyarov et al. (1969) reported quadrannulate *Orobdella* species from Primorsky Krai, Russia, as *Orobdella whitmani*. Although they did not describe the detailed internal anatomy of the specimen, the photograph of the ventral surface of their specimen (fig. 1 in their paper) clearly shows that the male gonopore opened in the furrow of XI/XII and the female gonopore was at XIII a1. Thus, the number of annuli between the gonopores was 4 + 1/2. This characteristic is not identical to those of the other known quadrannulate *Orobdella* species. There is a strong possibility that the quadrannulate *Orobdella* species distributed in Primorsky Krai is an undescribed species. Primorsky Krai is located at the same latitude as Hokkaido, Japan. Clarifying the taxonomic status and phylogenetic position of *Orobdella* in Primorsky Krai will help to reveal the species diversity and the evolutionary history of the genus *Orobdella*.

### Key to the known species of the genus Orobdella

**Table:** 

1	Mid-body somites more than quadrannulate	2
–	Mid-body somites quadrannulate	5
2	Mid-body somites sexannulate	3
–	Mid-body somites octannulate	*Orobdella octonaria* Oka, 1895
3	Pharynx reaching to XVI	4
–	Pharynx reaching to XIV, gonopores separated by 1/2 + 7 + 1/2 annuli	*Orobdella ijimai* Oka, 1895
4	Gonopores separated by 8 annuli	*Orobdella dolichopharynx* Nakano, 2011
–	Gonopores separated by 9 annuli	*Orobdella shimadae* Nakano, 2011
5	Color yellowish	6
–	Color grayish blue or brown	7
6	Gonopores separated by 1/2 + 5 annuli, gastroporal duct bottle-shaped	*Orobdella tsushimensis* Nakano, 2011
–	Gonopores separated by 1/2 + 4 + 1/2 annuli, gastroporal duct bulbiform	*Orobdella whimtani* Oka, 1895
7	Color grayish blue	8
–	Color brown, gonopores separated by 1/2 + 4 + 1/2 annuli	*Orobdella koikei* sp. n.
8	Gonopores separated by 2/3 + 4 + 1/3 annuli, gastroporal duct tubular, but bulbous at junction with gastropore	*Orobdella esulcata* Nakano, 2010
–	Gonopores separated by 6 annuli, gastroporal duct simple tubular	*Orobdella kawakatsuorum* Richardson, 1975

## Supplementary Material

XML Treatment for Orobdella koikei

XML Treatment for Orobdella kawakatsuorum
